# Deoxycholic Acid Mitigates Necrotic Enteritis Through Selective Inhibition of Pathobionts and Enrichment of Specific Lactic Acid Bacteria

**DOI:** 10.3390/pathogens14070688

**Published:** 2025-07-13

**Authors:** Melanie A. Whitmore, Jiaqing Guo, Dohyung M. Kim, Jing Liu, Isabel Tobin, Guolong Zhang

**Affiliations:** Department of Animal and Food Sciences, Oklahoma State University, Stillwater, OK 74078, USA; melanie.whitmore@okstate.edu (M.A.W.); jiaqing.guo@okstate.edu (J.G.); dohyung.kim@okstate.edu (D.M.K.); jing.liu12@okstate.edu (J.L.); isabel.tobin@okstate.edu (I.T.)

**Keywords:** deoxycholic acid, necrotic enteritis, microbiota, lactic acid bacteria, pathobionts, *Clostridium perfringens*

## Abstract

Necrotic enteritis (NE), caused by *Clostridium perfringens*, poses significant economic challenges to the global poultry industry. The widening ban on in-feed antibiotics in livestock production underscores the need for alternative strategies to combat NE. Deoxycholic acid (DCA), a secondary bile acid, has shown promise in NE mitigation. However, its protective mechanism remains largely unexplored. A total of 120 newly hatched, male Cobb broilers were randomly divided into four treatments to investigate the impact of DCA on host response and intestinal microbiome in both healthy and NE-infected chickens. The results demonstrated that the dietary supplementation of 1.5 g/kg DCA significantly improved animal survival, reversed growth inhibition, and alleviated intestinal lesions (*p* < 0.01). Furthermore, DCA selectively inhibited the NE-induced proliferation of *C. perfringens* and other pathobionts such as *Escherichia* and *Enterococcus cecorum*. Concurrently, DCA markedly enriched dominant lactic acid bacteria like *Lactobacillus johnsonii* in both the ileum and cecum of NE-infected chickens. However, DCA had a marginal effect on the jejunal transcriptomic response in both mock- and NE-infected chickens. Therefore, we conclude that DCA protects chicken from NE mainly through the targeted inhibition of pathobionts including *C. perfringens*, with minimum impact on the host. These findings elucidate the protective mechanisms of DCA, supporting its development as a promising antibiotic alternative for NE mitigation.

## 1. Introduction

The misuse and overuse of antibiotics in humans and livestock animals are primary drivers of antimicrobial resistance [1,2], which is projected to become the leading cause of global human mortality by 2050 [1]. Consequently, the subtherapeutic use of medically important antibiotics for growth promotion and disease prevention in livestock has been banned in an increasing number of countries; however, this ban has inadvertently increased the risk of infections in animals [3]. For example, necrotic enteritis (NE), a major poultry disease, has seen a marked rise in prevalence following the ban on in-feed antibiotics [4]. Additionally, antibiotic-free production systems have reported a sharp increase in NE incidence [5,6]. Therefore, there is an urgent need for antibiotic-alternative strategies to combat antimicrobial resistance, safeguard human and animal health, and ensure the efficiency of animal production.

*Clostridium perfringens*, a Gram-positive bacterium, is the etiological agent of NE. Infection with *C. perfringens* often follows exposure to *Eimeria* species, which damage the intestinal lining, trigger an inflammatory response, and disrupt the gut microbiota, creating an environment conducive to the proliferation of *C. perfringens* and subsequent disease manifestation [7]. Clinical NE presents with symptoms such as diarrhea, depression, and intestinal lesions, with mortality rates reaching up to 50% [8]. NE-induced lesions are confined to the small intestine, and in severe cases, present as extensive necrosis of the mucosal lining. Subclinical NE is more prevalent and associated with reduced growth performance and feed efficiency due to intestinal damage [3].

Various antibiotic alternatives are being explored to manage NE in poultry, including acidifiers, probiotics, and essential oils; however, few are nearly as efficacious and cost-effective as in-feed antibiotics [3,8]. Recently, deoxycholic acid (DCA) was found to be promising in mitigating NE [9,10,11,12]. DCA is a secondary bile acid derived from a primary bile acid, cholic acid, by intestinal bacteria belonging mainly to the genera *Eubacterium* and *Clostridium* [13,14]. DCA possesses both antibacterial and immunomodulatory effects [14,15,16]. Specifically, it is shown to induce the expression of the genes for host defense peptides and barrier function in chicken jejunal explants [12]. Additionally, DCA supplementation is associated with reduced inflammation in chickens with NE [11]. However, the impact of DCA on the intestinal microbiota remains largely unexplored.

In this study, we examined the effect of DCA on the intestinal mucosal transcriptional response and microbiome of healthy and NE-affected chickens. Our findings indicate that DCA significantly reduces the levels of *C. perfringens* and other pathobionts such as *Escherichia* and *Enterococcus cecorum* in NE-infected chickens. Moreover, DCA promotes the enrichment of beneficial lactobacilli in the ileum. However, a minimum response was observed in the jejunal transcriptome of both healthy and NE-infected chickens. These results shed light on the protective mechanisms of DCA, contributing to the development of DCA as a promising antibiotic alternative for the control and prevention of NE.

## 2. Materials and Methods

### 2.1. Chicken Trial

To study the influence of DCA on the intestinal microbiome of healthy and NE-infected chickens, a total of 120 day-of-hatch, apparently healthy, male Cobb broiler chicks were obtained from Cobb-Vantress Hatchery (Siloam Springs, AR, USA) and housed in an environmentally controlled room under standard management as recommended by Cobb-Vantress. Chickens were tagged with wing bands, weighed individually, and randomly assigned to one of four treatments: mock-infected control, DCA, NE, and DCA + NE. Each treatment consisted of 30 chickens in three pens with 10 birds per pen. Animals had ad libitum access to a standard nonmedicated corn-soybean meal mash starter diet (21% crude protein) that meets or exceeds the nutrient requirements of the National Research Council (NRC) recommendations [17] (Table S1). The diet was supplemented with or without 1.5 g/kg of DCA (Cayman Chemical, Ann Arbor, MI, USA) throughout the entire trial. This dose of DCA was chosen based on earlier findings [9,10,11,12].

A co-infection model was used to induce NE as previously described [12,18]. Briefly, chickens were orally challenged with 5 × 10^3^ sporulated oocysts of *E. maxima* M6 strain (kindly provided by Dr. John R. Barta, University of Guelph, Guelph, ON, Canada) in 1 mL saline on d 10 after overnight fasting. To encourage oocyst recycling, water was sprayed onto wood shavings twice daily at 9 AM and 5 PM on d 10 and 11. On d 14, approximately 4–5 × 10^8^ CFU of *C. perfringens* strain Brenda B, carrying *netB* and *tpeL* toxin genes (kindly provided by Dr. Lisa Bielke at North Carolina State University, Raleigh, NC, USA), was orally inoculated in 2 mL fluid thioglycollate (FTG) broth (Thermo Fisher Scientific, Waltham, MA, USA) after overnight fasting. Mock-infected control and DCA-only chickens were inoculated with 1 mL saline and 2 mL FTG broth on d 10 and 14, respectively.

All birds were weighed individually on d 10 and 17, and survival was monitored twice daily till the end of the trial on d 17. Chickens that were unable to stand, move, eat, or drink were euthanized by CO_2_ asphyxiation prior to death to minimize undue pain. All surviving animals were sacrificed on d 17, and the jejunum and ileum were scored for gross lesions of NE using a 6-point scoring system as described [19]. Approximately 0.5 g of the digesta from the proximal ileum and 0.2 g of the cecal digesta were aseptically collected randomly from 12 animals per treatment and four animals per pen. Additionally, approximately 1 cm of distal jejunal tissue was collected randomly from six animals per treatment and two animals per pen. The samples were immediately snap-frozen in liquid nitrogen and stored at −80 °C until further analysis. All animal procedures were approved by the Institutional Animal Care and Use Committee of Oklahoma State University under Protocol number IACUC-21-65.

### 2.2. Bacterial 16S rRNA Gene Sequencing and Data Analysis

ZR Fecal DNA MicroPrep and MiniPrep Kit (Zymo Research, Irvine, CA, USA) were used to isolate microbial genomic DNA from the ileal and cecal digesta, respectively. DNA concentration and quality were assessed using a NanoDrop^TM^ One Spectrophotometer (Thermo Fisher Scientific). High quality DNA was shipped on dry ice to Novogene (Beijing, China). PCR amplification of the V3–V4 region of bacterial 16S rRNA gene was performed using primers (341F: CCTAYGGGRBGCASCAG and 806R: GGACTACNNGGGTATCTAAT). DNA libraries were prepared using NEBNext^®^ Ultra™ Library Prep Kit (New England Biolabs, Ipswich, MA, USA), followed by PE250 deep sequencing on the NovaSeq 6000 system (Illumina, San Diego, CA, USA), generating a minimum of 30,000 raw sequencing reads per sample.

The downstream bioinformatic analysis of raw short sequencing reads was conducted as previously described [12,18,20,21,22]. Briefly, demultiplexed raw reads without adaptors and primers were analyzed using QIIME 2 v2023.9 [23]. The Deblur algorithm v2023.9.0 [24] was used to denoise by removing low-quality reads to produce filtered amplicon sequence variants (ASVs). ASVs appearing in less than 5% of samples were removed from further analysis. ASVs were classified using the Greengene2 database [25]. The top 150 ASVs were further confirmed and reclassified, if necessary, based on the EzBioCloud 16S database (v2023.08.23) [26].

The analysis and visualization of α- and β-diversities of the microbiota composition were conducted in R v4.3.2 [27], utilizing the ‘phyloseq’ package v1.46.0 [28]. The number of ASVs, Pielou’s evenness index, and Shannon index were used to calculate and display richness, evenness, and overall α-diversity, respectively. The β-diversity was determined using weighted and unweighted UniFrac distances [29]. The differential abundance of bacteria between groups was determined using the linear discriminant analysis (LDA) effect size (LEfSe) v1.1.01 [30], with the all-against-all multiclass analysis using *p* < 0.05 and a logarithmic LDA score of ≥3.0 as the threshold. ANCOM-BC2 [31] was further employed to reveal differential abundance of individual bacteria among four groups of animals.

### 2.3. RNA Sequencing and Data Analysis

Distal jejunal tissues were collected from six chickens per treatment on d 17 and lysed in RNAzol RT (Molecular Research Center, Cincinnati, OH, USA) for total RNA extraction. RNA concentrations were quantified using a Nanodrop^TM^ One Spectrophotometer (Thermo Fisher Scientific). High-quality RNA samples were shipped to Novogene (Sacramento, CA, USA) on dry ice for mRNA enrichment, fragmentation, reverse transcription, library preparation, and PE150 sequencing on Illumina NovaSeq 6000, generating at least 20 million raw sequencing reads per sample. After quality control, high-quality reads were mapped to the chicken reference genome (*Gallus gallus* GRCg7b) counted with featureCounts (v2.0.6) [32]. Genes with more than 10 counts in six samples per treatment were retained. DESeq2 (v1.42.0) [33] was employed to identify differentially expressed genes (DEGs) with a threshold of *p*-adjusted ≤ 0.01 and fold change ≥ 2. DEGs were then subjected to a Kyoto Encyclopedia of Genes and Genomes (KEGG) pathway enrichment analysis, with a *p*-adjusted < 0.05 considered significant.

### 2.4. Statistical Analysis

Statistical analysis and data visualization were implemented in GraphPad Prism 10 (GraphPad, La Jolla, CA, USA) or RStudio v1.2.1578 (RStudio, Boston, MA, USA). Statistical significance was measured using parametric or non-parametric methods, depending on the normality of the data based on the Shapiro–Wilk test and the homogeneity of variance based on the Levene’s test. Chicken body weight gain was compared among treatments using one-way analysis of variance (ANOVA) and post hoc Tukey’s test, while lesion scores, bacterial α-diversity, and relative abundance were compared using the Kruskal–Wallis test and post hoc Dunn’s test. Animal survival rates were compared using the log-rank test. Bacterial β-diversity was compared among groups using permutational multivariate analysis of variance (PERMANOVA) with 999 permutations using the ’vegan’ package v2.5.6 available in R. *p* < 0.05 was considered statistically significant.

### 2.5. Data Deposition

Raw sequencing reads of this study were deposited in the NCBI GenBank SRA database under BioProject ID: PRJNA1087128.

## 3. Results

### 3.1. Protection of Chickens from NE by DCA

**Figure 1:**
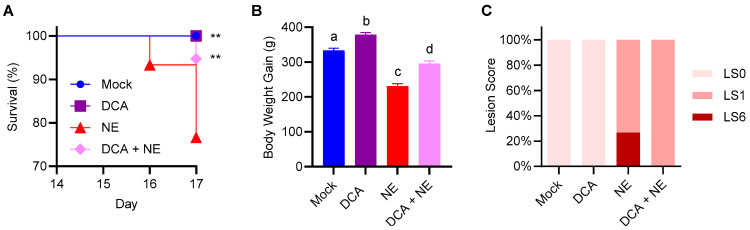
Protection of chickens from necrotic enteritis (NE) by deoxycholic acid (DCA). A total of 120 day-of-hatch male Cobb broilers were randomly assigned to one of four treatments (mock, DCA only, NE only, and DCA + NE) (*n* = 30 per treatment). Chickens were supplemented with or without 1.5 g/kg of DCA. On d 10 and 14, they were subjected to NE through sequential infections with *Eimeria maxima* and *C. perfringens*, respectively, or mock-infected on both days. (**A**) Animal survival (%) between d 14 and 17. Note that three groups (mock, DCA, and DCA + NE) are significantly different from the NE group (** *p* < 0.01) based on the log-rank test. (**B**) Body weight gains of surviving animals between d 10 and 17. Data shown are means ± SEM. Means with different superscripts denote statistical significance (*p* < 0.01) based on one-way ANOVA and post hoc Tukey’s test. (**C**) Frequency (%) of jejunal lesion scores of surviving animals on d 17.

A chicken trial was conducted to evaluate the protective efficacy of DCA against NE. As expected, two uninfected groups (mock-infected and DCA only) showed no mortalities. While 76.7% of infected chickens survived NE, dietary supplementation with 1.5 g/kg DCA significantly improved survival to 94.7% (*p* < 0.01), marking an 18% improvement (Figure 1A). Additionally, NE-infected chickens experienced significant weight loss, which was largely reversed with DCA supplementation (*p* < 0.001), resulting in a 27.8% increase in weight gain relative to NE-infected chickens (Figure 1B). However, DCA failed to fully restore weight gain following NE, as the body weight of DCA-supplemented NE chickens remained lower than that of mock-infected chickens (Figure 1B). Notably, among healthy chickens, DCA significantly enhanced body weight (*p* < 0.001; Figure 1B), suggesting its potential for growth promotion.

While no lesions were observed in two healthy groups, 26.7% of NE-infected chickens had a lesion score of six, while the remaining displayed very mild lesions with a score of one on a six-point scale (Figure 1C) [19]. Desirably, DCA obviously reduced lesion severity in NE-infected chickens, as evidenced by a lesion score of one for all chickens in this group (Figure 1C). These results showed clear benefits of DCA supplementation in improving the outcomes of NE in broiler chickens.

### 3.2. Impact of DCA on the Ileal Microbiome

To investigate the influence of DCA on the intestinal microbiome, ileal and cecal digesta from 12 surviving chickens in each treatment were subjected to DNA isolation and bacterial 16S rRNA gene sequencing. Following quality control, a total of 1,352,044 sequencing reads were generated, with an average of 29,392 ± 6979 sequences per ileal sample, while 1,318,297 raw reads were obtained, averaging 29,961 ± 5780 sequences per cecal sample. After filtering ASVs present in less than 5% of samples, 252 ASVs were identified in the ileum, and 360 ASVs in the cecum.

**Figure 2:**
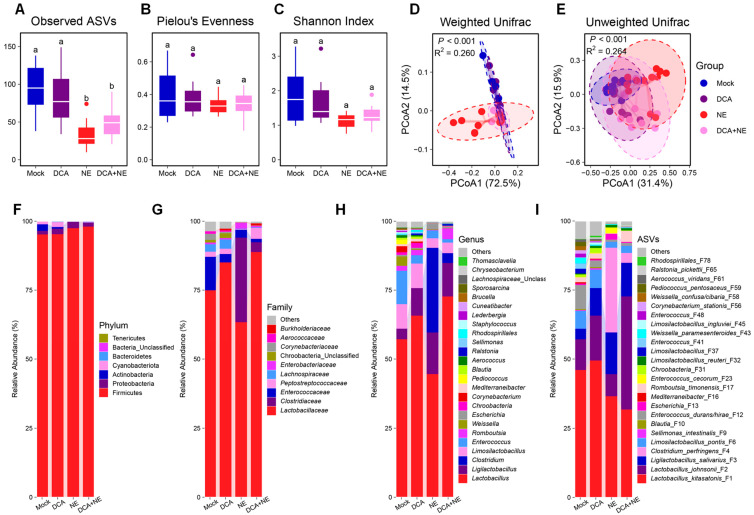
The diversity and composition of the ileal microbiota of healthy and NE-infected chickens in response to DCA supplementation. Day-of-hatch male Cobb broilers were supplemented with or without 1.5 g/kg of DCA. Two groups were subjected to NE, while the other two groups were mock-infected. The proximal ileal digesta was collected from 12 randomly selected surviving animals per group on d 17 and subjected to 16S rRNA gene sequencing. Observed ASVs (**A**), Pielou’s evenness index (**B**), and Shannon index (**C**) were calculated and visualized using box and whisker plots. Significance was measured using the Kruskal–Wallis test and post hoc pairwise Dunn’s test. Different superscripts denote significance (*p* < 0.05) in pairwise comparisons. Principal coordinates analysis (PCoA) plots of weighted (**D**) and unweighted UniFrac distances (**E**). Significance was determined using PERMANOVA. Relative abundances (%) of the top 7 phylum (**F**), 10 families (**G**), top 25 genera (**H**), and top 25 ASVs (**I**) in the ileal microbiota are shown.

The richness of the ileal microbiota, as indicated by the number of observed ASVs, was significantly reduced by NE, but failed to be restored by DCA supplementation (*p* < 0.05) (Figure 2A). However, the evenness (Figure 2B) and overall α-diversity (Figure 2C) of the ileal microbiota were comparable among the four treatments. Weighted and unweighted UniFrac distances indicated a significant alteration in the β-diversity of the ileal microbiota among groups (Figure 2D,E). Further pairwise comparisons revealed DCA had no significant impact on the β-diversity of the ileal microbiota of healthy chickens, while NE caused a substantial shift, and DCA supplementation failed to fully restore the microbiota to its normal state (Table S2).

Firmicutes accounted for 95 to 97% of the total bacterial population in the ileum (Figure 2F), with *Lactobacillaceae* (Figure 2G) and *Lactobacillus* (Figure 2H) being the predominant family and genus, respectively. At the species level, *Lactobacillus kitasatonis* (F1), *Lactobacillus johnsonii* (F2), and *Ligilactobacillus salivarius* (F3) represented 60–80% of all bacteria in the ileum (Figure 2I). While many bacterial populations experienced alterations in response to DCA or NE, the most obvious change occurred with *Clostridium perfringens* (F4), which was drastically increased by NE, but largely reversed with DCA supplementation.

**Figure 3:**
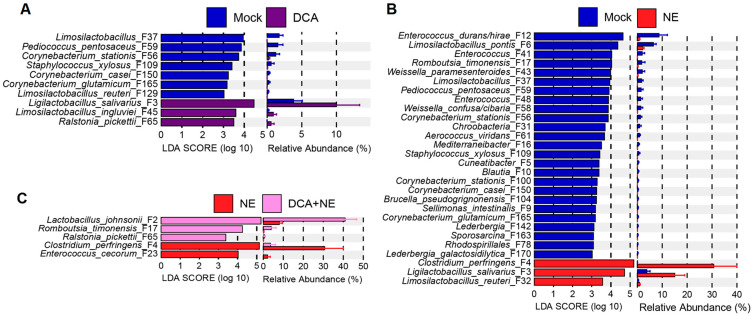
Differential enrichment of the top 50 ileal bacteria in healthy and NE-infected chickens in response to DCA supplementation. Day-of-hatch male Cobb broilers were supplemented with or without 1.5 g/kg of DCA. Two groups were subjected to NE, while the other two groups were mock-infected. The proximal ileal digesta was collected from 12 randomly selected surviving animals per group on d 17 and subjected to 16S rRNA gene sequencing. LEfSe analysis was performed between mock and DCA (**A**), between mock and NE (**B**), or between NE and DCA + NE chickens (**C**). The cut-off threshold was set at *p* < 0.05 and LDA score of ≥ 3.0. Each panel shows the LDA score of differentially abundant ASVs on the left, and their relative abundances (%) on the right.

An LEfSe analysis was further performed to identify differential enrichment of the ileal microbiota with the top 50 ASVs. DCA significantly enriched *L. salivarius* (F3), *Limosilactobacillus ingluviei* (F45), and *Ralstonia pickettii* (F65), while suppressing two other *Limosilactobacillus* species (F37 and F129) in healthy chickens (Figure 3A). *Pediococcus pentosaceus* (F59) and *Staphylococcus xylosus* (F109), as well as several species of *Corynebacterium* (F56, F150, and F165) were also suppressed by DCA in healthy chickens (Figure 3A). As expected, NE infection led to a significant increase in *C. perfringens* (F4) in the ileum (Figure 3B). Additionally, *L. salivarius* (F3) and *Limosilactobacillus reuteri* (F32) were significantly enriched in response to NE, while several other genera of lactic acid bacteria (LAB) including *Limosilactobacillus* (F6 and F37), *Weissella* (F43 and F58), *Pediococcus* (F59), and *Enterococcus* (F12, F41, and F48) were diminished (Figure 3B). DCA supplementation to NE-infected chickens significantly reduced *C. perfringens* (F4) from approximately 30% to 2%, while enriching *Lactobacillus johnsonii* (F2), *Romboutsia timonensis* (F17), and *Ralstonia pickettii* (F65) (Figure 3C). Furthermore, *E. cecorum* (F23), a pathobiont, was significantly diminished in NE-infected chickens supplemented with DCA (Figure 3C).

**Figure 4:**
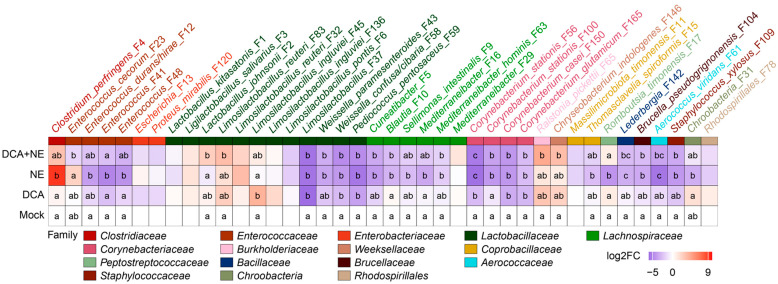
Heatmap illustrating the differential abundance of the top 40 ileal bacteria in healthy and NE-infected chickens in response to DCA. The family classifications of individual bacteria are indicated. Log2 fold changes (Log2FC) were calculated using mock-infected chickens as a common reference. Bacterial relative abundances are statistically different between treatments (*p*-adjusted < 0.05) by ANCOM-BC2 [31] if they share no letter in common within a column.

Among the top 40 most abundant bacteria in the ileum, two pathobionts, *C. perfringens* (F4) and *E. cecorum* (F23), bloomed in response to NE but were significantly reduced in NE-infected chickens supplemented with DCA (Figure 4). Interestingly, NE markedly suppressed three other presumably commensal *Enterococcus* species (F12, F41, and F48), with a tendency to be slightly reversed in response to DCA supplementation. Another common pathobiont, *Escherichia* (F13), and its related species *Proteus mirabilis* (F120) were not significantly affected by NE or DCA in the ileum.

Based on their response patterns, major ileal LAB could be separated into at least three groups: one group (F2, F3, and F45) enriched by DCA, one group (F6, F37, F43, F58, and F59) suppressed by DCA, and a third group (F1) largely unaltered (Figure 4). LAB abolished by DCA also experienced similar reductions in response to NE, and DCA supplementation had minimal impact on their abundances in NE-infected chickens (Figure 4). However, LAB enriched by DCA displayed different responses to NE. For example, *Lactobacillus johnsonii* (F2) tended to be suppressed by NE but was evidently enriched by DCA in NE-infected chickens (Figure 4).

### 3.3. Impact of DCA on the Cecal Microbiome

**Figure 5:**
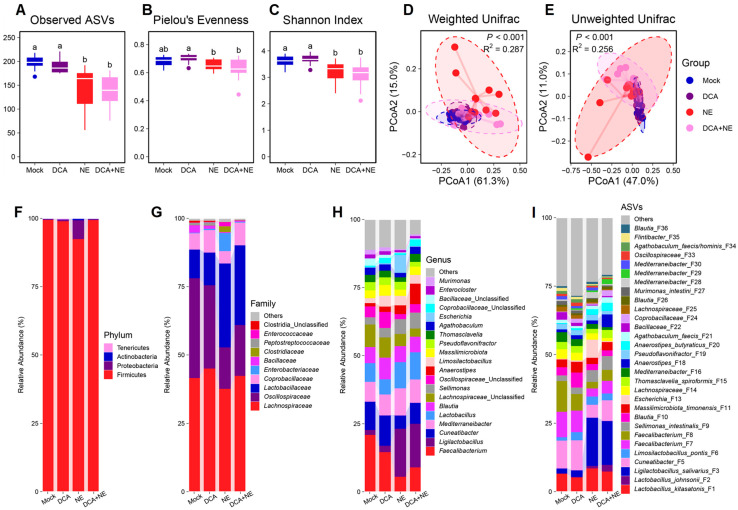
The diversity and composition of the cecal microbiota in healthy and NE-infected chickens in response to DCA supplementation. Day-of-hatch male Cobb broilers were supplemented with or without 1.5 g/kg of DCA. Two groups were subjected to NE, while the other two groups were mock-infected. The cecal digesta was collected from 12 randomly selected surviving animals per group on day 17 and subjected to 16S rRNA gene sequencing. Observed ASVs (**A**), Pielou’s evenness index (**B**), and Shannon index (**C**) were calculated and visualized using box and whisker plots. Significance was measured using the Kruskal–Wallis test and post hoc pairwise Dunn’s test. Different superscripts denote significance (*p* < 0.05) in pairwise comparisons. Principal coordinate analysis (PCoA) plots of weighted (**D**) and unweighted UniFrac distances (**E**). Significance was determined using PERMANOVA. Relative abundances of the top 4 phylum (**F**), 10 families (**G**), top 20 genera (**H**), and top 30 ASVs (**I**) in the cecal microbiota are shown.

In the cecum, NE infection significantly reduced the richness (Figure 5A), evenness (Figure 5B), and overall α-diversity (Figure 5C) of the microbiota, while DCA supplementation showed no discernible influence on either healthy or NE-infected chickens. Weighted UniFrac (Figure 5D) and unweighted UniFrac distances (Figure 5E) demonstrated significant differences in the β-diversity of the cecal microbiota among groups. Pairwise comparisons further indicated that DCA supplementation has negligible impact on the cecal microbiota composition of both healthy and NE-infected chickens (Table S3).

Compositionally, firmicutes were the predominant phylum in the cecum, constituting between 95% and 99% in total bacterial population (Figure 5F). *Lachnospiraceae*, *Oscillospiraceae*, and *Lactobacillaceae* accounted for approximately 85–90% of all bacteria across all treatment groups (Figure 5G). NE induced marked alterations in the three most abundant genera, *Faecalibacterium, Ligilactobacillus*, and *Cuneatibacter* (Figure 5H). At the ASV level, *L. salivarius* (F3) notably increased, while *Cuneatibacter* (F5) and *Faecalibacterium* (F7 and F8) decreased in response to NE, with *L. kitasatonis* (F1) largely unchanged (Figure 5I). DCA had a minimal impact on these bacteria in NE-infected chickens.

**Figure 6:**
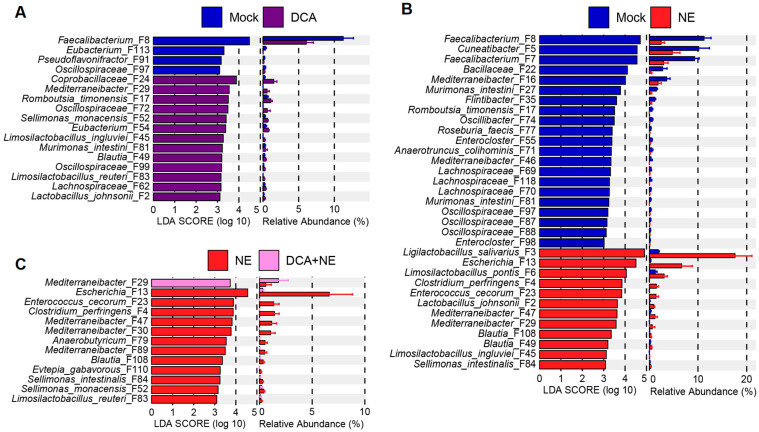
Differential enrichment of the top 100 cecal bacteria in healthy and NE-infected chickens in response to DCA supplementation. Day-of-hatch male Cobb broilers were supplemented with or without 1.5 g/kg of DCA. Two groups were subjected to NE, while the other two groups were mock-infected. The cecal digesta was collected from 12 randomly selected surviving animals per group on d 17 and subjected to 16S rRNA gene sequencing. LEfSe analysis was performed between mock and DCA (**A**), between mock and NE (**B**), or between NE and DCA + NE chickens (**C**). The cut-off threshold was set at *p* < 0.05 and LDA score of ≥ 3.0. Each panel shows the LDA score of differentially abundant ASVs on the left, and their relative abundances (%) on the right.

An LEfSe analysis of the top 100 ASVs in the cecum further revealed the differential enrichment of a large number of bacteria in response to DCA or NE (Figure 6). Among dominant bacteria, DCA suppressed *Faecalibacterium* (F8) from approximately 11% to 6%, while increasing several LAB species, such as *L. johnsonii* (F2)*, L. ingluviei* (F45), and *L. reuteri* (F83) (Figure 6A). Consistent with earlier studies [12,18], NE infection led to a drastic reduction in main short-chain fatty acid (SCFA) producers, such as *Faecalibacterium* (F7 and F8) and *Cuneatibacter* (F5), while multiple LAB species were significantly enriched (Figure 6B). Pathobionts such as *C. perfringens* (F4)*, Escherichia* (F13), and *E. cecorum* (F23) exhibited a bloom in NE-infected chickens (Figure 6B), which were largely abolished by DCA (Figure 6C). For example, DCA reduced *Escherichia* from approximately 7% to less than 1% following NE infection (Figure 6C).

**Figure 7:**
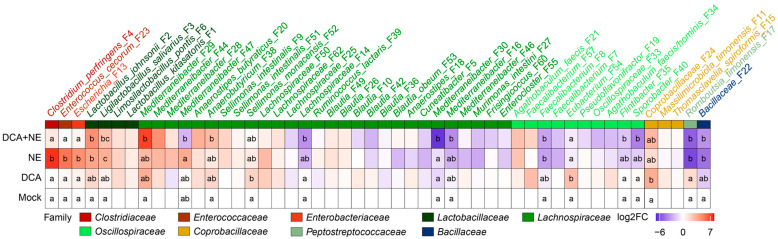
Heatmap illustrating the differential abundance of the top 50 cecal bacteria in healthy and NE-infected chickens in response to DCA. The family classifications of individual bacteria are indicated. Log2 fold changes (Log2FC) were calculated using mock-infected chickens as a common reference. Bacterial relative abundances are statistically different between treatments (*p*-adjusted < 0.05) by ANCOM-BC2 [31] if they share no letter in common within a column.

Among the top 50 most abundant cecal bacteria, all three major pathobionts (*C. perfringens* F4, *Escherichia* F13, and *E. cecorum* F23) significantly bloomed following NE infection but were substantially reduced by DCA (Figure 7), implying the selective antibacterial properties of DCA. Among the dominant LAB in the cecum, *L. johnsonii* (F2) and *L. salivarius* (F3) showed marked increases in response to NE infection and remained elevated following DCA supplementation. However, *L. kitasatonis* (F1) and *L. pontis* (F6) were minimally affected by either NE or DCA. Additionally, many dominant SCFA-producing bacteria within *Lachnospiraceae* and *Oscillospiraceae* families were diminished in response to NE, but DCA generally exerted a lesser impact on those bacteria in both healthy and NE-infected chickens (Figure 7).

### 3.4. Influence of DCA on the Intestinal Transcriptional Response

**Figure 8:**
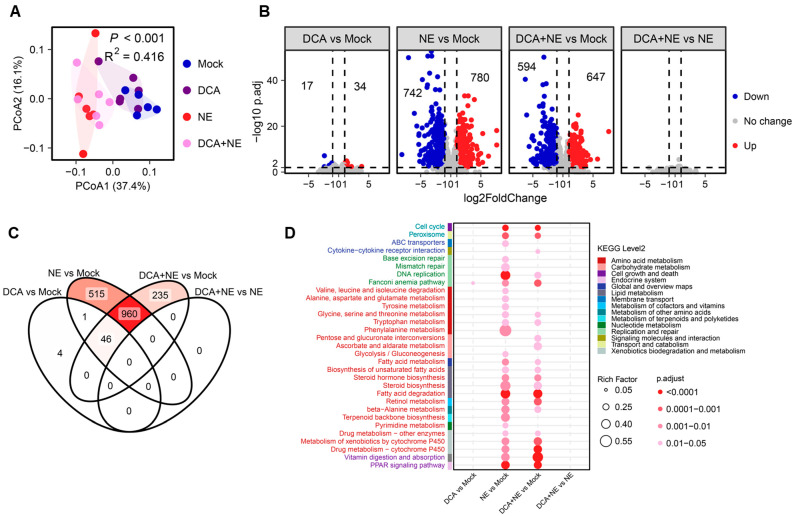
The influence of DCA on jejunal transcriptional response. Six distal jejunal tissue samples were collected from each group on d 17 following an NE challenge and subjected to RNA sequencing. (**A**) Principal coordinate analysis (PCoA) plot of the Bray–Curtis distances of the jejunal transcriptome among four groups of chickens. (**B**) Volcano plots of differentially expressed genes (DEGs) among different pairs of chicken groups. DEGs were identified with a threshold of *p*-adjusted ≤ 0.01 and fold change ≥ 2. The number of up- and downregulated genes are indicated in each plot. (**C**) Venn diagram of DEGs among different pairs of chicken groups. (**D**) KEGG pathway enrichment analysis of DEGs, with *p*-adjusted < 0.05 considered significant.

RNA sequencing of the chicken jejunal tissues were performed to assess the impact of DCA supplementation on the host response under both healthy and NE conditions. DCA supplementation had minimum impact on the jejunal transcriptome of mock- and NE-infected chickens, while NE caused a substantial transcriptomic shift (Figure 8A). DESeq2 analysis [33] revealed that only 51 genes were significantly altered in DCA-supplemented, mock-infected chickens, while NE caused the differential expression of 1522 genes, with 742 genes upregulated and 780 downregulated (Figure 8B). DCA supplementation failed to change the jejunal gene expression in NE-infected chickens, with no DEGs identified (Figure 8B). Among all DEGs, 46 genes were commonly regulated by DCA in both mock- and NE-infected chickens, while 960 genes were commonly altered in NE-infected chickens with and without DCA supplementation (Figure 8C). A KEGG pathway enrichment analysis further indicated that NE substantially altered jejunal transcriptional responses, including pathways related to DNA replication and repair, and amino acid and lipid metabolism; however, DCA supplementation had minimal impact on the host response in both mock- and NE-infected chickens (Figure 8D).

## 4. Discussion

Bile acids, such as DCA, play an important role not only in lipid digestion and adsorption, but also in maintaining intestinal homeostasis through their antibacterial and immunomodulatory properties [14,15,16]. The supplementation of DCA to broiler chickens has demonstrated protective effects against NE, as evidenced by increased survival, improved body weight gain, and reduced severity of intestinal lesions [9,10,11]. Mechanistically, DCA exhibits direct antibacterial activity against *C. perfringens*, with the capacity to suppress inflammatory responses, particularly through the inhibition of cyclooxygenase-2 signaling [11]. Additionally, DCA enhances the expression of multiple host defense peptide genes and several key genes involved in barrier function [12]. However, its impact on the intestinal microbiome remains unexplored. This study aimed to elucidate the influence of DCA supplementation on the intestinal microbiome of chickens under both healthy and NE conditions.

NE causes intestinal damage and inflammation, leading to the proliferation of facultative or aerotolerant pathobionts such as *C. perfringens*, *E. cecorum*, and *Escherichia* [12,18,34]. Although *C. perfringens* is mainly responsible for NE, the roles of other pathobionts in exacerbating the condition remain unclear. Interestingly, many of these pathobionts also proliferate in human inflammatory enteric disorders, such as inflammatory bowel diseases [35] and necrotizing enterocolitis [36]. Strategies to control these pathobionts are currently being explored for their potential utility against both disorders [35,36].

Our findings demonstrate that DCA supplementation at 1.5 g/kg provides significant protection to chickens from NE, without fully restoring the intestinal microbiota to its healthy state. Intriguingly, the microbiome of DCA-supplemented chickens more closely resembles that of infected chickens rather than that of healthy ones. A striking feature of DCA supplementation is its targeted elimination of NE-induced overgrowth *C. perfringens*, *E. cecorum*, and *Escherichia*. This selective inhibition is likely due to the direct antibacterial activity of DCA against *C. perfringens* and *Enterococcus* through its detergent-like properties, resulting in increased membrane permeability and leakage of cellular contents [37]. Even at concentrations as low as 0.1 mM, DCA is reported to eradicate *C. perfringens* in vitro [11], while the dietary supplementation of 1.5 g/kg accumulates DCA to concentrations of 1–5 mM in the ileum [9,10], effectively eradicating *C. perfringens* in the intestinal tract. The reduction in *E. cecorum* may also be attributed in part to DCA’s direct antibacterial action, as evidenced by the substantial killing of *Enterococcus* by DCA at 0.5%, equivalent to 1.27 mM, in vitro [38]. However, *E. coli* generally exhibits low sensitivity to DCA [39], with a minimum inhibitory concentration (MIC) of approximately 200 mM or 80 mg/mL [40]. Hence, the observed decrease in *Escherichia* is unlikely due to direct bacterial killing. Rather, it is likely the result of reduced *C. perfringens*-triggered intestinal mucosal damage and inflammation, resulting in the reduced proliferation of *Escherichia.* Attenuation in intestinal inflammation is associated with reduced proliferation of *E. cecorum* [41]. Therefore, a diminishment in *E. cecorum* is likely attributed to both targeted killing by DCA and suppressed intestinal inflammation.

Interestingly, DCA supplementation leads to the enrichment of several major LAB in both the ileum and cecum of healthy and NE-infected chickens. For example, *L. johnsonii* constitutes approximately 8% of the total bacteria in the ileum of healthy chickens, which rises to approximately 40% in NE-infected chickens upon DCA supplementation, with NE infection having a negligible impact. Similarly, *L. salivarius*, another prevalent LAB, increases from 4% in the ileum of healthy chickens to 10% in DCA-supplemented chickens and maintains similar levels in NE-infected chickens. However, *L. kitasatonis*, a dominant LAB, is a notable exception. It accounts for approximately 48% and 6% of total bacteria in the ileum and cecum, respectively, but appears to be minimally affected by either DCA or NE infection in both the ileum and cecum.

While there is a lack of research on the effects of DCA on healthy intestinal microbiota, our previous findings revealed that DCA supplementation enriched many LAB species in the ileum of NE-infected broilers, including group A Lactobacillus (*L. acidophilus*, *L. crispatus*, and *L. gallinarum*), *L. pontis*, and *Ligilactobacillus animalis*, while no other major LAB species show an obvious increase in either the ileum or cecum [12]. The discrepancy is likely due to differences in the severity of the NE model employed in both studies, as NE severity and intestinal damage can significantly influence the differential enrichment of LAB species [18]. In this current study, NE infection led to severe score-6 lesions in 22% of chickens without dietary intervention, while all NE-infected chickens exhibited only mild score-1 lesions with DCA supplementation. In our earlier study, approximately 50% of NE-infected chickens showed severe score-5 or -6 lesions, while 22% infected chickens had severe score-6 lesions following 1.5 g/kg DCA supplementation [12]. Nevertheless, several major LAB species showed enrichment in response to DCA supplementation across both studies. Additionally, a similar enrichment of *Limosilactobacillus* and *Ligilactobacillus* has been observed in rats following 12 weeks of oral administration with DCA and salicylate [42].

The enrichment of major LAB species mediated by DCA likely provides added protection against NE infection in chickens. Many LAB have shown promise as probiotics for NE control, with *L. johnsonii*, *L. salivarius*, and *L. reuteri* being among the most extensively studied candidates [43]. For instance, the oral administration of *L. johnsonii* has been demonstrated to enhance growth performance and reduce ileal damage in broilers with NE [44,45,46]. Furthermore, certain strains of *L. salivarius* and *L. reuteri* possess individual anti-*C. perfringens* activity and alleviate NE severity in broilers when combined [47]. Additionally, oral inoculation with four LAB species has shown potential in mitigating NE [48].

However, it remains unclear why many LAB species are enriched by DCA in both healthy and NE-infected chickens, despite their known sensitivity to DCA [39]. While the MICs of DCA against LAB such as *L. salivarius*, *L. gasseri*, and *L. reuteri* are generally less than 1 mM [49,50], LAB tolerance to bile acids can vary significantly among species and strains [39]. Therefore, the enriched LAB strains in response to DCA are likely resistant to mM concentrations of DCA, particularly in the ileum.

SCFA-producing bacteria, such as those in the *Lachnospiraceae* and *Oscillospiraceae* families, play a crucial role in maintaining host health and managing disease [51]. These bacteria often diminish during inflammatory intestinal disorders and are thus being explored for therapeutic purposes [52]. Similarly, many of these bacteria are significantly reduced in both the ileum and cecum of NE-infected chickens; however, DCA supplementation generally has no major impact on them. For example, *Faecalibacterium* is a prominent SCFA producer in the cecum, and its reduction is associated with many intestinal inflammatory disorders in humans [53]. NE infection significantly suppresses *Faecalibacterium* in the cecum, and DCA has a negligible effect on its regulation in both healthy and infected chickens. Although the impact of *Faecalibacterium* on NE is currently unknown, its administration has been found to alleviate intestinal inflammation in mice [53], suggesting it may be beneficial in mitigating NE. Similarly, several other major SCFA producers, such as *Cuneatibacter* (F5) and *Mediterraneibacter* (F16), are reduced in NE-infected chickens and remain diminished in response to DCA supplementation. These results reinforce the notion that DCA protects against NE by selectively eliminating pathobionts without fully restoring the intestinal microbiota to its healthy state.

DCA is known to be cytotoxic and damage DNA, and higher levels of DCA in the colon are associated with higher incidences of cancer [54], which is consistent with our RNA sequencing results showing the enrichment of pathways involved in DNA replication and repair. While DCA offers clear benefits in alleviating NE and dietary inclusion at 1.5 g/kg appears to enhance short-term weight gain in healthy broiler chickens, further investigation is warranted regarding its long-term effects on animal growth performance and overall physiology and health. Our recent study demonstrating synergy in host defense peptide synthesis and NE alleviation between DCA and another intestinal bacterial metabolite, butyrate, suggests the potential for using reduced amounts of DCA for disease management while minimizing potential undesirable side effects [12].

In this study, we observed that DCA exerted minimal influence on the host intestinal mucosal transcriptomic response in both healthy and NE-infected chickens despite DCA’s well-documented immunomodulatory effects [14,15,16]. The discrepancy between our findings and the known immunomodulatory properties of DCA may be attributed to differences in DCA concentration. The dietary supplementation of 1.5 g/kg of DCA could result in an accumulation of DCA to antibacterial concentrations of 1–5 mM in the ileum [9,10], whereas the optimal concentration for exerting immune regulatory activity, such as dendritic cell activation and host defense peptide gene induction, is less than 0.1 mM [12,55]. Nevertheless, we cannot rule out the possibility of DCA regulating host immunity systemically following dietary supplementation. It is plausible that μM concentrations of DCA could reach the circulation and impact the host immunity and physiology, contributing to the protection of chickens from NE. Future studies should measure the concentrations of DCA and its metabolites in the blood upon dietary supplementation.

## 5. Conclusions

In summary, the dietary supplementation of DCA provides robust protection against NE in chickens, highlighting its potential for the control and prevention of this disease. The main protective mechanisms of DCA appear to involve selective inhibition of pathobionts such as *C. perfringens*, *Escherichia*, and *E. cecorum*, while promoting various LAB species with minimum influence on the host. These findings suggest that DCA and DCA-enriched LAB offer promising antibiotic-free strategies for mitigating NE.

## Data Availability

Raw sequencing reads of this study were deposited in the NCBI GenBank SRA database under BioProject ID: PRJNA1087128.

## Supplementary Materials

The following supporting information can be downloaded at: https://www.mdpi.com/article/10.3390/pathogens14070688/s1, Table S1: Composition (%) of the broiler starter diet; Table S2: Statistical significance of weighted and unweighted UniFrac distances in the ileal microbiota of chickens in response to necrotic enteritis (NE) or deoxycholic acid (DCA); Table S3: Statistical significance of weighted and unweighted UniFrac distances in the cecal microbiota of chickens in response to necrotic enteritis (NE) or deoxycholic acid (DCA).

## Abbreviations

The following abbreviations are used in this manuscript:

DCA	Deoxycholic acid
DEG	Differentially expressed gene
FTG	Fluid thioglycollate
GO	Gene ontology
KEGG	Kyoto Encyclopedia of Genes and Genomes
LAB	Lactic acid bacteria
LEfSe	Linear discriminant analysis effect size
LDA	Linear discriminant analysis
NE	Necrotic enteritis
SCFA	Short-chain fatty acid
