# Supplementary material for: Deoxycholic Acid Mitigates Necrotic Enteritis Through Selective Inhibition of Pathobionts and Enrichment of Specific Lactic Acid Bacteria
Source: Pathogens. 2025 Jul 13;14(7):688. doi: 10.3390/pathogens14070688 (PMC12300365; doi:10.3390/pathogens14070688)
Supplement: Supplementary file 1 [file pathogens-14-00688-s001.zip › pathogens-3735503-supplementary.pdf]

Table S1. Composition (%) of the broiler starter diet

Ingredient	Composition (%)
Corn	52.8
Soybean meal	39.7
Soybean oil	3.5
Dicalcium phosphate, 18.5%	2.0
Limestone	1.1
Salt, >96%	0.5
DL-Methionine,	0.2
Threonine, 98%	0.1
Poultry premix*	0.3
Total	100.0
AME_n (kcal/kg)	2987.6
Crude protein (%)	21.5

* Poultry vitamin-mineral premix (NB-3000, Nutrablend, Neosho, Missouri, USA) supplied the following per kg of diet: manganese, 0.02 mg; zinc, 0.02 mg; iron, 0.01 mg; copper, 0.0025 mg; iodine, 0.0003 mg; selenium, 0.00003 mg; folic acid, 0.69 mg; choline, 386 mg; riboflavin, 6.61 mg; biotin, 0.03 mg; vitamin B6, 1.38 mg; niacin, 27.56 mg; pantothenic acid, 6.61 mg; thiamine, 2.20 mg; menadione, 0.83 mg; vitamin B12, 0.01 mg; vitamin E, 16.53 IU; vitamin D3, 2,133 ICU; vitamin A, 7,716 IU.

Table S2. Statistical significance of weighted and unweighted UniFrac distances in the ileal microbiota of chickens in response to necrotic enteritis (NE) or deoxycholic acid (DCA).

Group	Mock	DCA	NE	DCA + NE
Mock		*P = 0.123*<br>*R^2= 0.068*	*P = 0.002*<br>*R^2= 0.289*	*P = 0.002*<br>*R^2= 0.239*
DCA	P = 0.351<br>R^2= 0.045		*P = 0.002*<br>*R^2= 0.233*	*P = 0.008*<br>*R^2= 0.136*
NE	P = 0.018<br>R^2= 0.195	P = 0.018<br>R^2= 0.225		*P = 0.008*<br>*R^2= 0.140*
DCA + NE	P = 0.018<br>R^2= 0.159	P = 0.103<br>R^2= 0.092	P = 0.018<br>R^2= 0.227	

Note: A total of 120 day-of-hatch male Cobb broilers were supplemented with or without 1.5 g/kg of DCA. Two groups were subjected to NE, while the other two groups were mock-infected. The proximal ileal digesta samples (*n* = 12 per group) were collected on d 17 and subjected to DNA isolation and bacterial 16S rRNA gene sequencing. The lower triangle represents weighted UniFrac distances between groups. The upper triangle (italicized) displays unweighted UniFrac distances. Statistical significance was determined using PERMANOVA with 999 permutations.

Table S3. Statistical significance of weighted and unweighted UniFrac distances in the cecal microbiota of chickens in response to necrotic enteritis (NE) or deoxycholic acid (DCA).

Group	Mock	DCA	NE	DCA+NE
Mock		*P = 0.008*<br>*R^2= 0.096*	*P = 0.002*<br>*R^2= 0.224*	*P = 0.002*<br>*R^2= 0.269*
DCA	P = 0.104<br>R^2= 0.096		*P = 0.002*<br>*R^2= 0.217*	*P = 0.002*<br>*R^2= 0.241*
NE	P = 0.003<br>R^2= 0.334	P = 0.003<br>R^2= 0.280		*P = 0.149*<br>*R^2= 0.075*
DCA+NE	P = 0.020<br>R^2= 0.243	P = 0.036<br>R^2= 0.166	P = 0.165<br>R^2= 0.083	

Note: A total of 120 day-of-hatch male Cobb broilers were supplemented with or without 1.5 g/kg of DCA. Two groups were subjected to NE, while the other two groups were mock-infected. Cecal digesta samples (*n* = 12 per group) were collected on d 17 and subjected to DNA isolation and bacterial 16S rRNA gene sequencing. The lower triangle represents weighted UniFrac distances between groups. The upper triangle (italicized) displays unweighted UniFrac distances. Statistical significance was determined using PERMANOVA with 999 permutations.
